# Effect of an online resourcefulness training in improving psychological well-being of front-line medical staff: a quasi-experimental study

**DOI:** 10.1186/s40359-022-00920-7

**Published:** 2022-09-15

**Authors:** Dandan Zhang, Yitong Jia, Yongjun Chen, Ge Meng, Xinqi Zhuang, Li Chen, Dongmei Wang, Yin-Ping Zhang

**Affiliations:** 1 School of Nursing, Xi’an Jiaotong University Health Science Center, No. 76, Yanta West Road, Xi’an, 710061 Shaanxi China; 2 Institute of Clinical Research, Affiliated Nanhua Hospital, Hengyang Medical School, University of South China, 336 Dongfeng Road, Zhuhui District, Hengyang, 421002 Hunan China

**Keywords:** Anxiety, COVID-19, Depression, Epidemics, Psychology

## Abstract

**Background:**

The global COVID-19 pandemic is still not under effective control, and strong workplace supports with comprehensive mental health interventions are urgently needed to help medical staff effectively respond to the pandemic. This study aimed to verify the effect of an online resourcefulness training program on the resourcefulness, and psychological variables of front-line medical staff working in the COVID-19 isolation ward.

**Design:**

A pre-test and post-test quasi-experimental design with control group was employed.

**Participants:**

A total of 60 participants working in two isolation wards were recruited via convenience sampling. The two isolation wards were randomly assigned to the control group (isolation ward 1, *n* = 30) and the intervention group (isolation ward 2, *n* = 30).

**Intervention:**

The participants were trained online by video conferences and WeChat. The control group received conventional training (e.g., psychological training, psychological counseling), while the intervention group received a 4-h online resourcefulness training. Both groups learned updated guidelines of COVID-19 simultaneously via video conference. The primary outcomes (resourcefulness, anxiety, depression and coping styles) and the secondary outcome (psychological resilience) were measured before intervention and three time points after intervention.

**Results:**

After the intervention and one week after the intervention, the resourcefulness, resilience, and positive response scores of the participants in the intervention group were significantly higher than those of the control group. The anxiety and negative response scores in the intervention group were significantly lower than those of the control group (all *p* < 0.05).

One month after the intervention, the scores of resourcefulness, tenacity, and positive response of the intervention group were higher than those of the control group (all *p* < 0.05). Repeated measures analysis of variance showed that the two groups of participants had statistically significant changes in the time-based effect and group-based effect in resourcefulness, resilience, anxiety scores and coping styles (*p* < 0.01).

**Conclusion:**

The results showed that our online resourcefulness training can significantly improve the resourcefulness, resilience, and positive response scores and effectively reduce anxiety and depression scores of front-line medical staff. This demonstrates that online resourcefulness training would be an effective tool for the psychological adjustment of front-line medical staff in fighting against COVID-19.

## Introduction

With the spread of the pandemic, the number of patients with COVID-19 has increased sharply, and the front-line medical staff who have the most contact with patients are also facing a high risk of infection. Reports showed that in the first few months of the pandemic (until early April 2020), 3387 medical staff in China were infected with COVID-19, of which 23 (approximately 0.7%) died from the infection. [1, 2] At the same time, in Italy, approximately 20% of medical staff were infected [3]. In the United States, medical staff accounted for 11% of all reported cases. [4]

The front-line medical staff fighting against the COVID-19 pandemic not only face a high risk of infection, heavy workload but also face an unfamiliar working environment and the pressure of mutual adaptation to unfamiliar team members. Sometimes they also need to deal with the negative emotions of the patients. Front-line medical staff are more prone to adverse psychological stress in such a special environment [5]. According to studies during the COVID-19 pandemic, the anxiety, depression, fear, and stress of front-line medical staff working in high-risk areas were significantly higher than those in low-risk areas [6–9]. The global COVID-19 pandemic is still not under effective control and strong workplace support, the mental health interventions are urgently needed to help medical staff effectively respond to the pandemic.

Some international organizations, such as the World Health Organization, have issued documents suggesting that during the COVID-19 pandemic, psychological services such as targeted psychological counseling and interventions should be provided to improve the psychological well-being of the medical staff [10–12]. At present, many researchers have provided suggestions or interventions to improve the mental health of medical staff during the COVID-19 period, such as providing social support, psychological services, incentives, financial support and adequate personal protection, creating a safe environment and enhancing the capabilities of medical staff through education or training [13–15]. There were also remote interventions related to the mental health of the front-line medical staff such as using mindfulness interventions based on electronic devices to reduce psychological distress. [16, 17] However, most of these interventions mainly focused on a single level, whether it was the personal level of medical staff or the social level. Few studies have integrated the personal, social and special environmental to carry out comprehensive psychological interventions on front-line medical staff. Teaching medical staff how to integrate themselves into the culture of a new organization and how to get help from organizations is expected to improve problem-solving abilities of medical staff, thereby reducing their stress.

Resourcefulness includes personal resourcefulness (learned resourcefulness) and social resourcefulness. It refers to the ability of an individual to perform daily affairs independently (personal resourcefulness) and the ability to obtain help from others when the individual is unable to perform daily affairs independently (social resourcefulness) [18, 19]. Studies have shown that individuals with high resourcefulness scores can effectively use problem-solving strategies, reduce or eliminate the self-harm of stress, alleviate immediate needs, and control negative emotions [20–22]. Zauszniewski J A et al. [23] found that personal resourcefulness can actively promote one’s quality of life through improving mental health, life satisfaction, adaptability, and positive behavior. Nurses with a high level of resourcefulness can effectively cope with work stress, and the higher level of resourcefulness, the lower level of depression and anxiety [20, 24]. They are better at controlling emotions, thinking positively and less being affected by negative elements from the work environment [25]. Although resourcefulness training provides an effective way for the personal and social skills training, it commonly used acceptable and feasible self-reinforcement methods for the majority of patients and caregivers, such as journals, texting, group processing, and voice recording, that are important for the effectiveness of resourcefulness training [26, 27]. Currently, intervention studies related to resourcefulness training have mainly focused on patients, disabled persons or their caregivers [28–30]. And very few intervention studies in medical staff were reported [20, 24, 31]. Thus, the aims of this study were to develop an online resourcefulness training program to train the first-line medical staff working in the COVID-19 isolation ward and to further verify the effect of this program on their resourcefulness and psychological variables.

## Methods

### Participants

The study was conducted in a designated hospital that intensively treats patients with COVID-19 in Hunan Province, China. The situation of COVID-19 pandemic gradually became most pressing with the rapid increase in newly confirmed cases during the study. Major public health emergency responses were activated across China. The government had put forward a series of extensive, stringent containment measures to prevent and control its spread, such as requiring designated hospitals to carry out the screening of fever patients, timely diagnosis, isolation and treatment of the new COVID-19 cases, and technical requirements for the protection of medical staff and patient management. Driven by the national spirit of selfless dedication, solidarity and cooperation and supported by the government, many medical staff voluntarily applied to work in the isolation ward. The local health commission selected qualified medical staff from applicants as needed and referred them to work in the isolation wards.

The participants in our study were recruited from two isolation wards of the infectious diseases department of the Hospital affiliated with University of South China via convenience sampling. The inclusion criteria were as follows: (a) willingness to participate; (b) working in the COVID-19 isolation ward for at least two weeks; and (c) signing informed consent. The exclusion criteria included (a) failure to fill out the questionnaire and (b) withdrawal from the study. A total of 60 medical staff working in two isolation wards were recruited to participate in the study. Two isolation wards were randomly assigned to the control group (isolation ward 1) and the intervention group (isolation ward 2) using a random number table. Each group consisted of 30 medical staff, including clinicians, nurses, rehabilitation specialists, and traditional Chinese medicine physicians.

### Design

This was a pre-test and post-test quasi-experimental study with a control group.

### Ethics

This study was approved by the Medical Ethics Committee of Affiliated Nanhua Hospital, University of South China (2020-ky-46) and was in accordance with the Helsinki Declaration of 1964. The informed consent obtained from participants was written. All participants were informed of voluntarily participating, they could withdraw from the study at any time. The participant's personal information is confidential and can only be accessed by the authors. All methods were performed in accordance with the relevant guidelines and regulations.

### Patient and public involvement statement

No patients were involved in this study. This study/article mainly focused on the effect of online resourcefulness training on the psychological variables and resourcefulness levels of medical staff.

### Training

From February 13 to 28, 2020, the medical staff in the control and intervention groups were trained according to the following program. Taking into account the working conditions of the isolation ward, lectures and training were conducted by video conferences. WeChat groups were created for the intervention group and control group. Participants were invited to join their own WeChat group through mobile phones or computers and could post or read information at any time during their work breaks, ensuring that the medical staff in both groups could obtain project-related information through the WeChat platform.

#### (1) The control group received conventional training

Firstly, an expert group was established with 6 members, including 1 medical expert, 1 nursing expert, 2 psychologists, 1 infection prevention and control specialist, and 1 observer. The criteria for selecting experts in our intervention was as follows: a) have obtained a senior professional title, b) have worked for more than 10 years in his/her profession. Observers were trained postgraduate medical students who were responsible for the information delivery and collection in WeChat group, as well as questionnaire surveys and daily contact with participants. Psychologists were responsible for providing psychological training and psychological counseling. During the isolation period, psychological training was intensively carried out for 1 h. After training, the participants were divided into 6 groups with 5 people in each group to receive a 30-min group psychological counseling for a total of 6 times. Psychologists also provided individual psychological counseling as needed. 3 participants in the control group received individual psychological counseling.

**Fig. 1:**
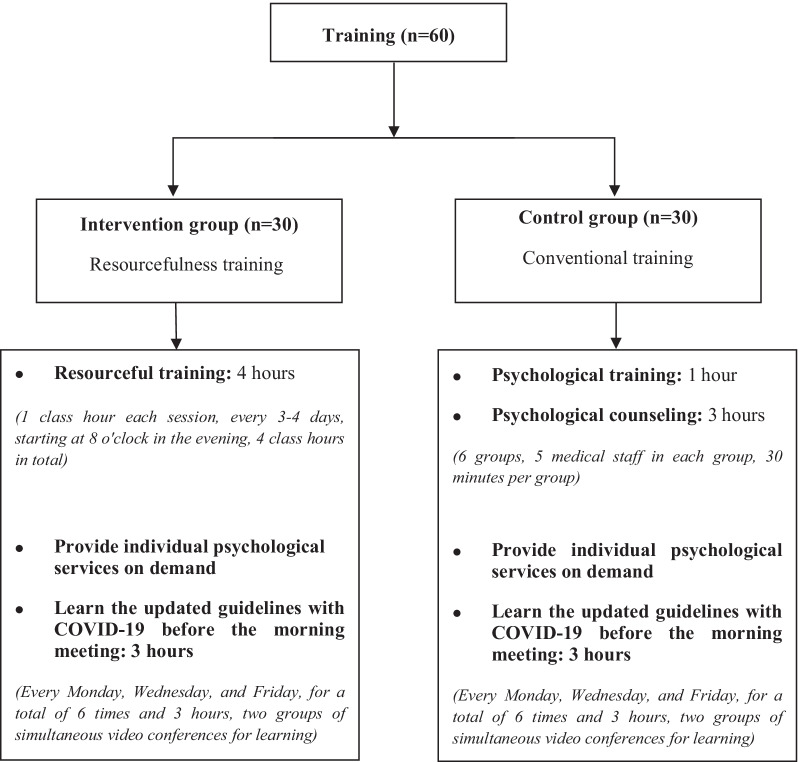
Training plan for medical staff in the two groups

Secondly, participants in the control group needed to study the updated guidelines on the diagnosis, treatment and nursing of patients with COVID-19, and the current nosocomial infection prevention and control guidelines for COVID-19. Remote guidance by experts, they studied the guidelines via video conference in the doctor's offices of the isolation ward for 30 mins before the morning meeting every Monday, Wednesday, and Friday (Fig. 1). The detailed training plan can be found in Appendix.

#### (2) The intervention group received online resourcefulness training

Firstly, a resourcefulness intervention team composed of 7 members was established, including 1 medical expert, 1 nursing expert, 2 psychologists, 1 infection prevention and control specialist, and 2 resourcefulness training members. Resourcefulness training members were professors who specialized in research related to resourcefulness training. They were responsible for implementing resourcefulness training, collecting data, delivering information in the WeChat group, and communicating with participants every day. Nursing expert was also trained in resourcefulness and was mainly responsible for solving nursing problems and assisting in the implementation of resourcefulness training.

Secondly, the intervention group carried out 4 periods of online resourcefulness training (Fig. 1). A training session was carried out every 3–4 days, starting at 8 o'clock in the evening, and lasting for 1 h each time. Considering that some participants were on duty at night, there were two training sessions for each period. Those who cannot participate in the first training session can join the second training session with the same content the next night. The training content includes emotional management skills, interpersonal communication, alternative experience, targeted incentives, positive self-concept, etc. The specific intervention methods and content are shown in Table 1. The detailed training plan can be found in Appendix.

**Table 1:** Contents of resourcefulness training

	The main frame	Secondary frame	Time	Contents	Methods
The first training	Personal resourcefulness	Redressive self-control	1 h	The trainer and participants get to know each other, and establish mutual trust and cooperation between colleagues	Explanations, demonstrations, on-site exercises team/group training and individual training
				Psychological experts explain the methods of emotional regulation, and organize medical staff to conduct emotional adjustment and relaxation training on site	
				Psychologists assess the participants who need psychological counseling, and conduct one-on-one counseling. Participants can also ask for help if they feel that they need psychological counseling during the work period	
The second training	Personal resourcefulness	Reformative self-control	1 h	Use targeted incentives: Let participants actively express their anxiety, stress, and worry, and encourage them to share their feelings with each other	Explanations,discussion, practice team/group training
				According to the participants’ expressions, 4 incidents of concern were found: ①How to effectively respond to the rescue of critically ill COVID-19 patients; ②Emergency treatment of occupational exposure incidents; ③How to do personal protection effectively and quickly; ④How to prevent the goggles from crushing the face or fogging	
				The intervention group was divided into 3 teams, and each team listed the 4 incidents in order of priority. If there are other problems, they can also list them. One-to-one correspondence with what they can solve by themselves, and if they can’t solve it, they are encouraged to seek help from the mentor team. Solve an incident, tick the matter, and give them a certain reward to encourage them	
The third training	Personal resourcefulness	Perceived self-efficacy	1 h	Instruct the team members to have a positive self-talk and help them form a positive self-concept. Allow them to share the voice diary in the WeChat group after getting off work every day, one or two sentences at a time, such as the happiest thing, the thing that I am most satisfied with, my strengths, and my progress in certain aspects, etc	Explanations, share, practice team/group training
				Alternative experience: organize medical team to watch positive news reporting that medical teams from all over the country have gathered in Hubei province to fight COVID-19, share successful experiences in isolation wards, show patients and their families expressing gratitude to medical team, etc., to provide self-confidence to the medical team	
The fourth training	Social resourcefulness	The use of formal and informal assistance	1 h	Tell the participants that the government, the hospital and the people in the region are supporting their work. Tell them about social donations (including protective materials, medical equipment, food, etc.) to alleviate participants’ worries	Explanations, communication team/group training and individual training
				The director of the department, the head nurse, or best friend, relative representatives, etc. were invited to video link with the participants to give positive encouragement and support to the team members	
				Instruct participants to use formal and informal assistance to solve problems when they encounter difficulties	

Thirdly, consistent with the control group, participants in the intervention group also studied the updated guidelines of COVID-19 via video conference before the morning meeting every Monday, Wednesday, and Friday. There were no differences in the study time and content between intervention group and control group.

In the intervention process, a combination of team/group training and individual training was used. COVID-19-related knowledge and training information were updated in a timely manner through the WeChat platform. Participants can actively log in during breaks to read messages, discuss, share or comment. During the study process, it was ensured that strict protective measures were taken in all personnel contacts, and the risk of infection caused by the project was strictly eliminated.

### Evaluation

The primary endpoints (resourcefulness, anxiety and depression and coping styles) and secondary endpoints (psychological resilience) were measured before and after the intervention, one week after the completion of the intervention, and one month after the completion of the intervention.

#### Main outcome indicators

① Resourcefulness: Use the Chinese version of the resourcefulness scale (RS) [18, 32]. The scale has two subscales reflecting personal (16 items) and social (12 items) resourcefulness. Each item was scored from 0 to 5, with a total score of 0 to 140 points. The higher the score, the more resourceful the respondent is. The Cronbach’s α of internal consistency of the scale was 0.825, indicating good reliability [18, 33]. ② Anxiety and depression: The Self-Rating Anxiety Scale (SAS) and the Self-Rating Depression Scale (SDS), which were both designed by Zung [34], were used to evaluate the anxiety and depression state of the participants. The scales were widely used and had high reliability and validity. ③ Coping style: Use the Simple Coping Style Questionnaire (SCSQ), developed by Folkman and Lazarus [35], which was translated and revised into Chinese by Xie [36]. It includes two dimensions, positive response (12 items) and negative response (8 items), with responses ranging from "Never" to "Very often" (equivalent to scores of 0–3). The higher the score, the more likely the respondent is to choose this coping style. The scale was credible. [37, 38]

#### Secondary outcome measures

The secondary outcome was psychological resilience. The Chinese version of Connor and Davidson’s Resilience Scale was applied to measure resilience. [39, 40] It contains three factors: Tenacity, Strength, and Optimism, with a total of 25 items. The items were rated on a 5-point Likert scale from "not true at all" to "true nearly all the time" (equivalent to scores of 0–4). The scale was evaluated and showed good reliability and validity. [40, 41]

### Statistical analysis

All data were checked by two researchers and imported into SPSS 18.0 for statistical analysis. The scores before and after the intervention of the two groups were compared. Student’s *t-*test was employed to test the data conforming to a normal distribution. The Wilcoxon rank-sum test was applied if the data did not conform to a normal distribution. Repeated measures analysis of variance was used to measure the scores of each scale at 3 time points after the intervention. A *p-value* less than 0.05 was considered statistically significant.

## Results

### (1) Participant characteristics

There were 30 patients in the intervention and control groups, respectively. Table 2 presents the baseline characteristics of the participants. The differences in gender, age, clinical experience, highest degree, profession, professional title and department of work between the two groups were not statistically significant (*p* > 0.05).

**Table 2:** Participant characteristics in two groups (*n* = 60)

Variable	Control group n (%)	Intervention group *n* (%)	*χ*2	*p-value*
*Gender*				
Male	6 (20.0)	10 (33.3)	1.364	0.243
Female	24 (80.0)	20 (66.7)		
*Age (years)*				
18–25	7 (23.3)	7 (23.3)	1.476	0.688
26–29	11 (36.7)	10(33.3)		
30–39	9 (30.0)	12 (40.0)		
≥ 40	3 (10.0)	1 (3.3)		
*Clinical experience (years)*				
≤ 5	9 (30.0)	12 (40.0)	1.344	0.719
5–9	9 (30.0)	10 (33.3)		
10–19	10 (33.3)	7 (23.3)		
≥ 20	2 (6.7)	1 (3.3)		
*Highest degree*				
Diploma/associate degree	6 (20.0)	4 (13.3)	1.518	0.468
Bachelor’s degree	18 (60.0)	16 (53.3)		
Master’s and Doctor’s degree	6 (20.0)	10 (33.3)		
*Professional title*				
Primary	14(46.7)	15 (50.0)	0.578	0.749
Intermediate	11 (36.7)	12 (40.0)		
Senior	5(16.7)	3(10.0)		
*Profession*				
Doctor	9(30.0)	8(26.7)	0.587	0.746
Nurse	18(60.0)	17(56.7)		
Others ^a^	3(10.0)	5(16.7)		
*Hospital department of work*				
Medical ward	8 (26.7)	13 (43.3)	2.076	0.722
Surgical ward	4 (13.3)	3(10.0)		
Emergency department	4 (13.3)	3(10.0)		
Infectious disease ward	5(16.7)	5(16.7)		
Others	9 (30.0)	6 (20.0)		

^a^ Chinese medicine doctors or rehabilitation specialists

### (2) Comparison of outcome variables of medical staff between intervention and control groups at different time points

Before the intervention, participants in the two groups were compared in their resourcefulness, resilience, anxiety, depression and coping styles scores, and the differences were not statistically significant (all *p* > 0.05). See Table 3.

**Table 3:** Outcome variables changes during four phases of study in the intervention and control groups

Variables	Group	Mean (SD)			
		Pre -intervention	Post -intervention	One week after intervention	One month after intervention
*Resourcefulness*					
Personal resourcefulness ^a^	Intervention	47.56(6.09)	53.10 (7.44)	54.93 (7.57)	57.67 (9.79)
	Control	47.23(6.11)	48.87 (5.37)	49.70 (5.19)	50.43 (6.45)
	*t*	− 0.212	− 2.528**	− 3.122**	− 3.380**
Social resourcefulness ^b^	Intervention	31.73(6.39)	38.17 (5.25)	40.40 (6.00)	42.60 (7.17)
	Control	31.40(7.08)	33.63(7.36)	34.20 (7.60)	36.30 (7.60)
	*t*	− 0.191	− 2.746**	− 3.506**	− 3.304**
Total scores ^c^	Intervention	79.30(9.50)	91.27 (10.80)	95.33 (10.80)	100.27 (15.78)
	Control	78.63(8.72)	82.50 (9.9)	83.90 (13.10)	86.73(11.12)
	*t*	− 0.283	− 3.276**	− 4.241**	− 3.840**
*Psychological variables*					
*Anxiety* ^d^	Intervention	43.40(5.58)	37.80 (5.12)	34.30 (5.49)	32.43 (6.90)
	Control	43.97(5.46)	40.90 (4.76)	39.77 (5.49)	35.63 (6.72)
	*t*	0.398	2.430**	3.857**	1.820
*Depression* ^e^	Intervention	45.17(5.21)	41.10 (4.42)	38.97 (4.85)	34.83 (7.32)
	Control	44.93(5.78)	43.80 (5.90)	41.30 (6.41)	38.27 (8.97)
	*t*	− 0.164	2.006*	1.590	1.625
*Coping Style*					
Positive response ^f^	Intervention	26.87 (3.99)	34.30 (3.99)	34.30 (3.99)	36.37 (5.87)
	Control	27.27 (4.21)	30.10 (4.25)	30.10 (4.25)	31.63 (4.60)
	*t*	0.378	− 3.947**	− 3.947**	− 3.475**
Negative response ^g^	Intervention	24.53 (2.26)	19.40 (3.46)	17.46 (3.34)	15.10 (3.32)
	Control	24.80 (3.17)	23.03 (3.17)	21.53 (3.79)	20.03 (3.77)
	*t*	0.376	4.242**	4.407**	5.380**
*Resilience*					
Tenacity ^h^	Intervention	9.73 (2.26)	12.40 (1.99)	13.47 (1.83)	14.10 (2.07)
	Control	9.37 (1.97)	10.53 (1.94)	11.27 (1.76)	12.10 (2.22)
	*t*	− 0.670	− 3.673**	− 4.741**	− 3.068**
Strength ^i^	Intervention	23.90 (4.11)	28.50 (3.13)	31.73 (3.82)	33.87 (6.12)
	Control	24.33 (4.10)	25.80 (3.62)	28.13 (4.42)	32.27 (5.64)
	*t*	0.409	− 3.090**	− 3.376**	− 1.053
Optimism ^j^	Intervention	31.83 (3.98)	35.77 (4.36)	38.50 (4.46)	40.77 (7.80)
	Control	31.07 (3.86)	31.97 (3.66)	34.07 (3.58)	30.07 (7.70)
	*t*	− 0.757	− 3.655**	− 4.244**	− 1.349

Sample size of the two groups: *n* = 30 (in intervention group at 3 time points); *n* = 30 (in control group at 3 time points)

SD: standard deviation, using *t* (*t* test)

^*^p < 0.05, ** *p* < 0.01

a Pr model: (group) F = 12.633, *p* < 0.05;(time) *F* = 6.673, *p* < 0.05;(group × time interaction) *F* = 1.646, *p* > 0.05

b Sr model: (group) F = 11.750, *p* < 0.05;(time) *F* = 20.341, *p* < 0.05;(group × time interaction) *F* = 1.566, *p* > 0.05

c Ts model: (group) F = 17.416, *p* < 0.01;(time) *F* = 17.349, *p* < 0.05;(group × time interaction) *F* = 2.251, *p* > 0.05

d A model: (group) F = 90.45, *p* < 0.05;(time) *F* = 34.969, *p* < 0.05;(group × time interaction) *F* = 2.024, *p* > 0.05

e D model: (group) F = 3.493, *p* > 0.05;(time) *F* = 44.470, *p* < 0.05;(group × time interaction) *F* = 0.359, *p* > 0.05

f Pr model: (group) F = 17.991, *p* < 0.05;(time) *F* = 9.354, *p* < 0.05;(group × time interaction) *F* = 0.205, *p* > 0.05

g Nr model: (group) F = 27.937, *p* < 0.05;(time) *F* = 51.115, *p* < 0.05;(group × time interaction) *F* = 1.679, *p* > 0.05

h T model: (group) F = 24.790, *p* < 0.05;(time) *F* = 18.719, *p* < 0.05;(group × time interaction) *F* = 0.197, *p* > 0.05

i S model: (group) F = 8.175, *p* < 0.05;(time) *F* = 42.082, *p* < 0.05;(group × time interaction) *F* = 1.205, *p* > 0.05

j O model: (group) F = 11.137, *p* < 0.05;(time) *F* = 23.843, *p* < 0.05;(group × time interaction) *F* = 0.292, *p* > 0.05

After the intervention and 1 week after the intervention, the scores of resourcefulness, resilience, and positive response in the intervention group were significantly higher than those of the control group during the same period (all *p* < 0.05). The anxiety and negative response scores in the intervention group were significantly lower than those of the control group during the same period (all *p* < 0.05).

One month after the intervention, the scores of resourcefulness, tenacity and positive response of the intervention group were higher than those of the control group (all *p* < 0.05). Repeated measures analysis of variance showed that the two groups of participants had statistically significant changes in the time-based effect and group-based effect in resourcefulness, resilience, anxiety scores and coping styles (*p* < 0.01). The change in depression scores of participants in the intervention group was statistically significant in the time-based effect (*p* < 0.01). See Table 3.

## Discussion

In this quasi-experimental study, we analyzed the effects of online resourcefulness training on the resourcefulness, anxiety, depression, coping styles and psychological resilience of front-line medical staff fighting against COVID-19. A total of 60 medical staff working in isolation wards volunteered to participate in this training. To verify the effect of the training, we measured the outcome indicators before and after the intervention, one week after the intervention, and one month after the intervention. The results showed that our online resourcefulness training can produce a significant improvement in the scores of resourcefulness of the participants. At the same time, they will experience better psychological outcomes, including the lower SDS and SAS scores, a better level of resilience and more likely to choose positive coping style. These findings indicated that the online resourcefulness training would provide new evidence for improving the psychological adjustment ability, reducing negative emotions, and changing coping styles of front-line medical staff fighting against COVID-19.

Resourceful training is mainly used to cultivate the ability of personal (self-help) and social (seeking help) skills [23]. In our study, to improve the personal resourcefulness of the participants in the intervention group, they were trained to effectively respond to stressful events at work and guided to adopt new cognitive or behavioral approaches [42]. These measures enable the participants to change their original fixed thinking patterns and master the knowledge and skills of patient management as soon as possible. In addition, we shared and discussed alternative experience and used positive incentive measures to encourage and support them. To help them form positive self-concepts and enhance their sense of self-efficacy [43, 44], voice diaries were also shared to guide positive self-conversations [23]. To improve their social resourcefulness, we trained their abilities of using targeted incentives, that is, solving problems in accordance with their priorities. If the problems cannot be solved by themselves, they can actively seek help and social support [43, 45]. In addition, creating a good team atmosphere and cooperative relationship were also beneficial for improving social intelligence. After the intervention, the personal resourcefulness, social resourcefulness scores and total scores of the intervention group were significantly higher than those of the control group at 3 time points (*p* < 0.01), indicating that online resourcefulness training can significantly improve the resourcefulness of the front-line medical staff fighting against COVID-19.

Participants in both the intervention and control groups showed decreases in anxiety and depression scores after the intervention. However, participants in the intervention group had significantly lower anxiety and depression scores and higher resilience scores after the intervention than those in the control group (all *p* < 0.05), indicating the beneficial effects of online resourcefulness training on improving the psychological well-being of medical staff. Similar to our study, Zauszniewski JA et al. [46] found that improving personal resourcefulness can significantly reduce the anxiety and depression rates of diabetic patients by self-control. Some studies have indicated that personal resourcefulness is negatively correlated with the depression index. [24, 47] Ngai et al. [29, 48] guided psychological intervention with the concept of learned resourcefulness, which effectively improved the role competence and perinatal depressive symptoms of Chinese mothers. Yaqin Liu et al. [49] conducted staged group psychological interventions on breast cancer patients and found that resourceful training can effectively improve the negative emotions of women with breast cancer. The study by Irani, E et al. [30] showed that during the COVID-19 pandemic, home caregivers of adults with chronic and/or disabilities were under increasing stress. Resourcefulness can minimize the impact of stress on health outcomes. In contrast to other studies, the psychologists in our study conducted online resourcefulness training using the WeChat platform. The program trained the participants on how to manage emotions and relax themselves, encouraged them to express fear, worry and negative emotions, and conducted targeted psychological counseling, motivation, communication, and so on. These simple and convenient psychological intervention skills enable the participants to effectively decompress themselves and release negative emotions at work. The findings demonstrated that our online resourcefulness training would be an effective tool for improving the psychological well-being of medical staff during the COVID-19 epidemic.

Previous studies have shown that there is a significant correlation between coping styles and resourcefulness [26, 50, 51]. Patients usually choose negative coping styles when their resourcefulness level is relatively low [51]. Liang et al. [50] implemented 8 weeks of resourceful training for patients with coronary heart disease in the community and found that it can reduce patients' negative coping styles and enable patients to cope with the disease using a positive attitude. Huang et al. [26] improved the emotional control and coping ability of patients with nasopharyngeal carcinoma during radiotherapy through resourceful training. However, the subjects of these studies were patients. Our training program was designed for the front-line medical staff fighting against COVID-19. It enabled them to accumulate more coping skills in the work of the isolation ward, enhance their confidence and change their negative thinking into positive thinking. Resourceful training includes psychological adjustment, motivation, cooperation and communication, social support, etc., which can improve positive emotions and promote positive coping styles. The results of this study showed that the positive coping scores of the intervention group were significantly higher than those of the control group at 3 time points after intervention (*p* < 0.01), and the negative coping scores of the intervention group were significantly lower than those of the control group at 3 time points after the intervention (*p* < 0.01). It is suggested that resourcefulness training would help to change the coping style of front-line medical staff; the higher the resourcefulness score is, the higher the positive response scores.

The results (Table 3) showed that one month after the intervention, the anxiety and depression scores of the two groups gradually decreased compared with those in post-intervention, but there was no significant difference between the two groups (*p* > 0.05). The possible reasons were as follows: First, one week after the intervention, the medical staff in the two groups successfully completed their work and left the isolation ward after the handover with other teams, so the psychological pressure was relieved. Second, by successfully completing the work in the isolation ward, they gained experience in diagnosis and treatment related to COVID-19, received more social support and obtained extensive information so that the negative emotions of the participants were better released.

## Conclusion

Our study has developed an online resourcefulness training program to help improve the mental health of the front-line medical staff during the COVID-19 pandemic. We conducted four periods of online resourcefulness training for front-line medical staff in the isolation ward, and it significantly improved the resourcefulness and resilience scores of the medical staff under the COVID-19 pandemic. At the same time, the online resourcefulness training program was found to reduce the anxiety and depression scores of medical staff, help them respond more positively to complex, challenging work tasks. This demonstrates that our online resourcefulness training would be an effective tool for the psychological adjustment of front-line medical staff in fighting against COVID-19. Such training is expected to provide reference for front-line medical staff effective response to public health emergencies in the future.

### Limitations

There were some limitations in this study. First, the sample size of this study was relatively small, and since the participants were selected by the local health commission and transferred into two isolation wards, it was difficult to implement random sampling and grouping, which may have a certain impact on the generalizability of the results. The participants in our study had a broad range of education levels, medical specialties, and age range. The two isolation wards were randomly assigned to the control group and the intervention group. And there were no significant between-group differences for all baseline socioeconomic characteristics, indicating that the two groups were comparable and the results were acceptable. Second, due to the special working environment in the isolation ward, intervention training tended to be carried out in teams, and individual interventions, especially individual psychological training, were not flexible enough. Team training may influence the results to some extent. Third, the long-term effects of online resourcefulness training interventions need to be further observed.

## Data Availability

The datasets generated and/or analysed during the current study are not publicly available due to privacy restrictions but are available from the corresponding author on reasonable request.

## Appendix

### Training plan for the control group

**Table:**

Item	Time	Contents and class hours
Guidelines study Session 1	February 13 Friday 7:25–7:55	Lecture (0.5 h): clinical guidance for the management of COVID-19 patients, and the key points of nursing care
Psychological training	February 14 19:00–20:00	Lecture (1 h): common psychological counseling and emotion regulation methods, relaxation training
Guidelines study Session 2	February 16 Monday 7:20–7:50	Lecture (0.5 h): infection control guidance
Psychological counseling	February 17 16:30–17:00 19:30–20:00	The third group learning activity (0.5 h) The first group learning activity (0.5 h)
Guidelines study Session 3 Psychological counseling	February 18 Wednesday 7:30–8:00 17:00–17:30	Lecture (0.5 h): guidelines for the treatment and care of the COVID-19 patients in critically condition (I) The fifth group learning activity (0.5 h)
Psychological counseling	February 19 19:30–20:00	The second group learning activity (0.5 h)
Guidelines study Session 4 Psychological counseling	February 20 Friday 7:25–7:55 16:00–16:30	Lecture (0.5 h): guidelines for the treatment and care of the COVID-19 patients in critically condition (II) The sixth group learning activity (0.5 h)
Psychological counseling	February 21 18:30–19:00	The fourth group learning activity (0.5 h)
Guidelines study Session 5	February 23 Monday 7:20–7:50	Lecture (0.5 h): guidelines for traditional Chinese medicine treatment of COVID-19 Patients
Guidelines study Session 6	February 25 Wednesday 7:30–8:00	Lecture (0.5 h): learn the updated version of these guidelines
Note: During the work period, psychologists will assess members who need psychological counseling and conduct one-on-one counseling. Members can also seek help if they feel the need for psychological counseling while at work		

### Training plan for the intervention group

**Table:**

Item	Time	Content and class hours
Guidelines study Session 1	February 13 Friday 7:25–7:55	Lecture (0.5 h): clinical guidance for the management of COVID-19 patients, and the key points of nursing care
Resourcefulness training 1	February 14 20:00–21:00	Redressive self-control (1 h)
Resourcefulness training 1	February 15 20:00–21:00	Redressive self-control (1 h)
Guidelines study Session 2	February 16 Monday 7:20–7:50	Lecture (0.5 h): infection control guidance
Resourcefulness training 2	February 17 20:00–21:00	Reformative self-control (1 h)
Guidelines study Session 3	February 18 Wednesday 7:30–8:00	Lecture (0.5 h): guidelines for the treatment and care of the COVID-19 patients in critically condition (I)
Resourcefulness training 2	February 19 20:00–21:00	Reformative self-control (1 h)
Guidelines study Session 4	February 20 Friday 7:25–7:55	Lecture (0.5 h): guidelines for the treatment and care of the COVID-19 patients in critically condition (II)
Resourcefulness training 3	February 21 20:00–21:00	Perceived self-efficacy (1 h)
Resourcefulness training 3	February 22 20:00–21:00	Perceived self-efficacy (1 h)
Guidelines study Session 5	February 23 Monday 7:20–7:50	Lecture (0.5 h):guidelines for Chinese Medicine Treatment of COVID-19 Patients
Resourcefulness training 4	February 24 20:00–21:00	The use of formal and informal assistance (1 h)
Guidelines study Session 6 Resourcefulness training 4	February 25 Wednesday 7:30–8:00 20:00–21:00	Lecture (0.5 h):learn the updated version of these guidelines The use of formal and informal assistance (1 h)

There are two identical training sessions per session of resourcefulness training. Members on night shifts and those unable to attend the training may attend a second training of the same content the following evening
